# DiVenn: An Interactive and Integrated Web-Based Visualization Tool for Comparing Gene Lists

**DOI:** 10.3389/fgene.2019.00421

**Published:** 2019-05-03

**Authors:** Liang Sun, Sufen Dong, Yinbing Ge, Jose Pedro Fonseca, Zachary T. Robinson, Kirankumar S. Mysore, Perdeep Mehta

**Affiliations:** ^1^ Noble Research Institute, Ardmore, OK, United States; ^2^ College of Information Science and Technology, Hebei Agricultural University, Baoding, China

**Keywords:** Venn diagram, visualization, transcriptome data, KEGG, gene ontology, pathogen infection

## Abstract

Gene expression data generated from multiple biological samples (mutant, double mutant, and wild-type) are often compared *via* Venn diagram tools. It is of great interest to know the expression pattern between overlapping genes and their associated gene pathways or gene ontology (GO) terms. We developed DiVenn (Dive into the Venn diagram and create a force directed graph)—a novel web-based tool that compares gene lists from multiple RNA-Seq experiments in a force-directed graph, which shows the gene regulation levels for each gene and integrated KEGG pathway and gene ontology knowledge for the data visualization. DiVenn has four key features: (1) informative force-directed graph with gene expression levels to compare multiple data sets; (2) interactive visualization with biological annotations and integrated pathway and GO databases, which can be used to subset or highlight gene nodes to pathway or GO terms of interest in the graph; (3) Pathway and GO enrichment analysis of all or selected genes in the graph; and (4) high resolution image and gene-associated information export. DiVenn is freely available at http://divenn.noble.org/.

## Introduction

With the advance of high-throughput data technologies, huge amounts of gene expression data were generated without in-depth analysis. Several web-based visualization tools—for example, INVEX (Xia et al., 2013), ExAtlas (Sharov et al., 2015), and WebGIVI (Sun et al., 2017)—were successfully used in expression data analysis. However, systematically comparing more than two experiments is still challenging. It is especially challenging to visualize multiple experiments’ data along with integrated bioinformatics databases. Venn diagrams are widely used to compare gene lists among multiple experiments. GeneVenn (Pirooznia et al., 2007), Venny (Oliveros, 2015), and InteractiVenn (Heberle et al., 2015) are examples of web-based tools currently being used. However, they have significant limitations: (1) gene IDs cannot be linked to gene functions. No bioinformatics databases such as biological pathway and gene ontology (GO) can be integrated. (2) Gene expression levels cannot be displayed in the graph. (3) Common or unique genes, which are likely to be interesting in the Venn diagram cannot be extracted with gene expression value and gene function.

We provide an interactive web-based tool that will overcome the above-mentioned limitations and help biologists visualize their gene lists and generate biological hypotheses based on the integrated knowledge from biological pathway and GO databases. Using this tool, researchers cannot only compare and visualize gene lists, but also subset or highlight the gene nodes in the graph based on gene functions of interest. This tool is user friendly and can handle large amounts of input data by using a force-directed focus package.^1^ Users can extract and download important gene information from the result/information table and download the high resolution image for their publications.

## Features

**Figure 1:**
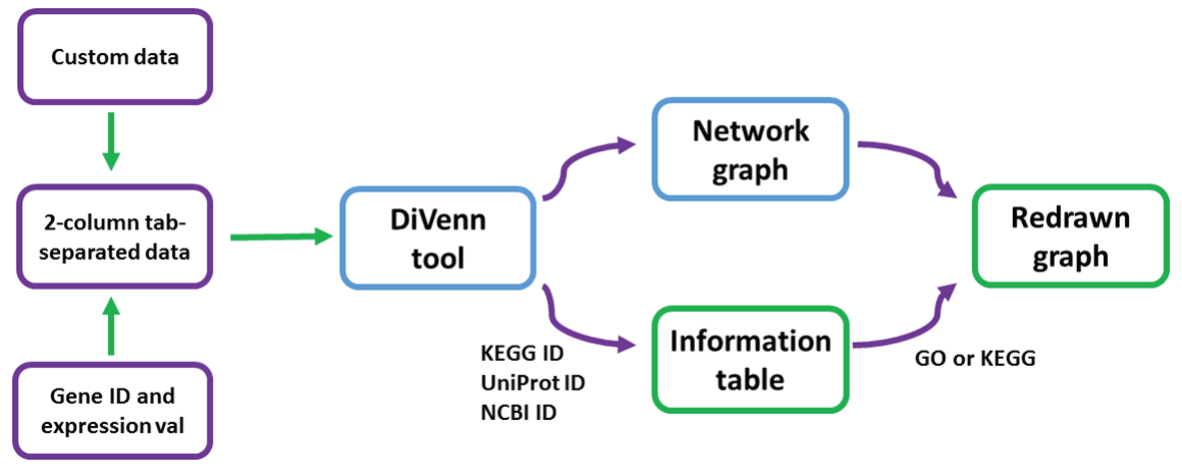
Flow chart of DiVenn. Two-column tab-separated gene ID and expression value data from supported 14 species can be used to redraw the graph based on the KEGG or GO information table.

DiVenn was developed using PHP, JavaScript, R, D3.js (Bostock et al., 2011), and MySQL database. The flow chart of the data visualization is depicted in Figure 1. DiVenn currently accepts two types of input data: (1) two-column tab-separated custom data. For example, gene ID and corresponding pathway data, transcription factors and their regulated downstream genes, and microRNAs and corresponding target genes. The second column must be “1” or “2”. (2) Gene expression data. The first column is gene IDs and the second column is gene regulation value. The gene regulation value should be obtained from differentially expressed (DE) genes. Users can select the cut-off value of fold change (for example, two-fold change) to define their DE genes. To simplify this gene regulation value, we require users to use “1” to represent upregulated genes and “2” to represent downregulated genes based on their own cut-off value of fold change. If users need to link their genes to the KEGG pathway (Kanehisa et al., 2019) or GO database, 14 model species with KEGG pathway and GO database available are supported in DiVenn. Currently, three types of gene IDs—KEGG gene IDs, Uniprot gene IDs (UniProt, 2008), and NCBI gene IDs (Benson et al., 2018)—are accepted for pathway analysis. All agriGO (Du et al., 2010; Tian et al., 2017) supported IDs are accepted for GO analysis by DiVenn. DiVenn allows users to compare and visualize up to eight gene lists in the network graph.

DiVenn has four major functions:

1. Comparison of gene lists from multiple experiments in the force-directed network graph with more integrated bioinformatics knowledge. As opposed to traditional Venn diagrams, which can show only the total number of overlapping genes among experiments, DiVenn can provide the information of gene expression regulation level, gene description, KEGG pathway, and GO terms.
2. Pathway and GO enrichment analysis of gene lists. DE genes from experiments with similar treatments are more likely to involve in the same pathway or GO terms. DiVenn applied the modified Fisher Exact test to enrich all or selected DE genes into significant KEGG pathways and GO terms similar to what have been used in well-known DAVID software (Jiao et al., 2012).^2^ The modified Fisher Exact test is constructed by using an R script (fisher.test).
3. Subset and highlighting of gene nodes of interest. To better visualize the input gene list and also subset the gene list to gene groups, especially to avoid “hairballs” when the gene list is too large, DiVenn has a function to subset gene lists to pathway and GO groups of interest in the force-directed network graph, or change the node shapes of genes of interest to square shapes in the original graph by using the redraw function in the information table. Accordingly, a new modified Fisher Exact test can be performed for the subset gene list.
4. High-resolution image and selected data in the graph export. The graph generated by DiVenn can be downloaded as portable network graphics (PNG) and scalable vector graphics (SVG) files. SVG images can be converted to high-resolution images.

## Results and Discussion

### Data Uploading and Processing

DiVenn accepts two-column tab-separated data for each experiment. The input data for each experiment is processed into JSON format and visualized *via* D3.js library. Each experiment is represented as one parent node (experiment node) with an automatically assigned color. All genes corresponding to the experiment are connected to the parental nodes *via* edges. If a gene node is clicked, the edges connecting to this gene node will be colored based on the expression values of this gene in the connected experiment node. GUI functions in the DiVenn graph provide the ability to: (1) Switch the gene label on and off. (2) Download images and data. (3) Change node color. (4) Display gene-related annotation, pathway, and GO information in a sortable table format.

### Database Integration

KEGG gene IDs and corresponding UniProt and NCBI gene ID maps, gene function description, and KEGG pathways are captured through KEGG API by self-written PYTHON scripts. GO ID, terms, and categories were downloaded from agriGO (Du et al., 2010; Tian et al., 2017). All these information were stored in the MySQL database and automatically updated in our systems. The KEGG pathway and GO database of 14 model species were integrated to DiVenn. Right-clicking a gene node in the force-directed graph provides options to show the gene name and gene-detailed information (gene description, KEGG pathway, and GO terms).

### Graph Redraw

DiVenn allows users to show all genes and their gene functions, associated KEGG pathways, KEGG maps, and GO terms in an information table. The modified Fisher Exact test is applied to all or selected genes in the graph. The *p*’s for pathways and GO terms are shown in the information table. This table can be sorted based on each column and is searchable by keywords of interest. Users can sort the table based on KEGG pathways or GO terms and can redraw gene nodes with square nodes in the graph when users need to highlight gene nodes of their interest, or subset gene nodes in a new graph based on the specific KEGG pathway or GO terms of interest, which will simplify the graph especially when a large gene list is being visualized. This knowledge is critical for users making biological hypotheses.

### Case Study

**Figure 2:**
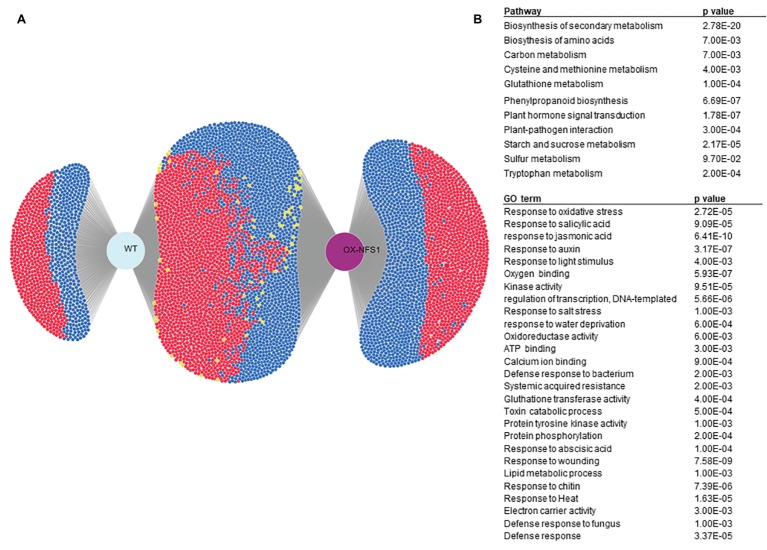
DiVenn case study showing differentially expressed genes between WT and *NFS1* overexpression line (OX-*NFS1*) in *Arabidopsis* upon bacterial pathogen inoculation. **(A)** Differentially expressed genes, up and downregulated (log2(FC)≤ −2 and ≥2) between 0 and 3 days, after pathogen inoculation in *Arabidopsis* for WT and OX-*NFS1* plants. Blue and red nodes denote downregulated and upregulated genes between different genotypes, respectively. Yellow nodes denote upregulation in one sample but downregulation in another. **(B)** Several pathways and GO terms significantly enriched (*p* < 0.05) in our dataset.

In order to demonstrate how DiVenn performs network, GO and pathway analysis using RNAseq data, we obtained a dataset consisting of all DE genes (log_2_ fold change ≥2 and ≤ −2) Figure 2A. This dataset consists of genes differentially expressed between 0 h (basal control) and 3 days after bacterial pathogen *Pseudomonas syringae* pv. tomato DC3000 inoculation in 5 week-old *Arabidopsis* plants for two different genotypes: wild-type (WT) and *NITROGEN FIXATION S (NFS1)-LIKE 1* overexpression line (OX-*NFS1*). We were also able to quickly select several pathways significantly enriched in our dataset from GO and KEGG pathway such as Oxidative stress (GO) and sulfur metabolism (pathway) (Figure 2B).

**Figure 3:**
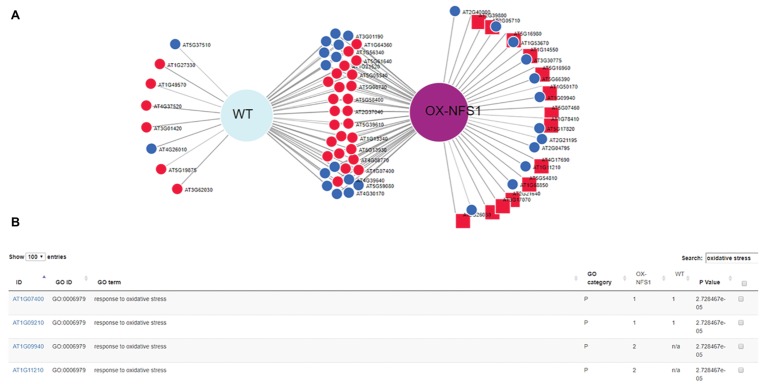
DiVenn subset analysis of genes obtained using redraw function from gene ontology (GO) search “oxidative stress” displaying 73 DE genes up and downregulated from OX-*NFS1* and WT. **(A)** Genes upregulated only for OX-*NFS1* are highlighted as square nodes *via* the redraw function. **(B)** Table consisting of genes from subset graphic GO search “oxidative stress” displaying DE genes between the two genotypes OX-*NFS1* and WT (just the four first rows out of 73) and GO category *p*’s. The numbers 1 and 2 refer to upregulated and downregulated genes, respectively, for each *Arabidopsis* gene accession number.

**Figure 4:**
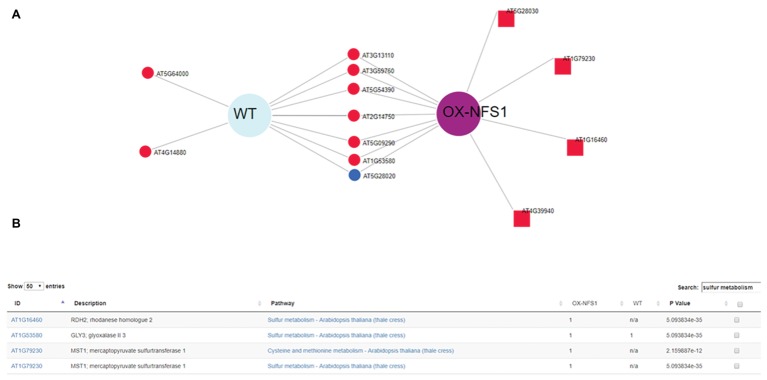
DiVenn subset analysis of genes obtained using redraw function from KEGG pathway analysis search “sulfur metabolism” showing 14 DE genes up and downregulated from OX-*NFS1* and WT. **(A)** Genes upregulated only for OX-NFS1 are highlighted as square nodes *via* the redraw function. **(B)** Table consisting of genes from subset graphic KEGG pathway search “sulfur metabolite” displaying DE genes between the two genotypes OX-*NFS1* and WT (just first four rows out of 14) and pathway *p*’s. The numbers 1 and 2 refer to upregulated and downregulated genes, respectively, for each *Arabidopsis* gene accession number.

DiVenn allowed us to quickly select only genes involved in the oxidative stress GO terms (GO:0006979) using a keyword search between DE genes from different experiments and generate a subset graph (Figure 3A). Oxidative stress genes are involved in several plant stress responses, including biotic and abiotic stresses as well as reactive oxygen species (ROS) generation. We created a subset graph of all 73 oxidative stress-related genes, significantly (*p* < 0.05) DE between both lines using the redraw function and we found more oxidative stress-related genes upregulated in the OX-*NFS1* line in comparison to WT upon bacterial pathogen infection (Figures 3A,B). Similarly, searches using “sulfur metabolism” as a keyword under the pathway menu allowed us to visualize all 14 significantly DE genes involved in sulfur metabolism from both genotypes (Figures 4A,B). We found more sulfur metabolism genes significantly upregulated in the OX-*NFS1* line compared to WT line upon pathogen treatment such as *MERCAPTOPYRUVATE SULFURTRANSFERASE 1* (*MST1*; AT1G79230) and *L-CYSTEINE DESULFHYDRASE 1* (*DES1*; AT5G28030) that are involved in the sulfur metabolic pathway. The above-mentioned examples are illustrative of the fact that plants under biotic stress go through an extensive transcriptional reprogramming affecting several genes from different pathways and organelles.

## Conclusion

We have successfully developed DiVenn, a web-based tool to visualize large gene lists from multiple experiments. This tool provides a promising approach for comparing multiple gene expression data sets. The integrated bioinformatics databases and interactive visualization graph will help biologists generate biological hypotheses.

## Author Contributions

LS conceived the original research plans. LS, YG, SD, and ZR developed this tool. JF and KM performed the case study. LS, JF, ZR, and PM wrote the manuscript. LS, KM, and PM edited the manuscript.

### Conflict of Interest Statement

The authors declare that the research was conducted in the absence of any commercial or financial relationships that could be construed as a potential conflict of interest.

## Footnotes

^1^http://ramblings.mcpher.com/Home/excelquirks/gassites/d3nodefocus

^2^https://david.ncifcrf.gov/content.jsp?file=functional_annotation.html#EXP
